# Barriers to and Facilitators of Key Stakeholders Influencing Successful Digital Implementation of Remote Monitoring Solutions: Mixed Methods Analysis

**DOI:** 10.2196/49769

**Published:** 2024-05-06

**Authors:** Fahad Mujtaba Iqbal, Ravi Aggarwal, Meera Joshi, Dominic King, Guy Martin, Sadia Khan, Mike Wright, Hutan Ashrafian, Ara Darzi

**Affiliations:** 1 Division of Surgery Imperial College London London United Kingdom; 2 West Middlesex University Hospital London United Kingdom; 3 Innovation Business Partner Chelsea and Westminster NHS Trust London United Kingdom

**Keywords:** implementation science, health plan implementation, mobile health, health care industry, stakeholder, COVID-19, remote monitoring, digital tools, digital health, pandemic, virtual wards, virtual ward, health care delivery, telemedicine, telehealth, wearables, wearable, technology, United Kingdom, UK, digital services

## Abstract

**Background:**

Implementation of remote monitoring solutions and digital alerting tools in health care has historically been challenging, despite the impetus provided by the COVID-19 pandemic. To date, a health systems–based approach to systematically describe barriers and facilitators across multiple domains has not been undertaken.

**Objective:**

We aimed to undertake a comprehensive mixed methods analysis of barriers and facilitators for successful implementation of remote monitoring and digital alerting tools in complex health organizations.

**Methods:**

A mixed methods approach using a modified Technology Acceptance Model questionnaire and semistructured interviews mapped to the validated fit among humans, organizations, and technology (HOT-fit) framework was undertaken. Likert frequency responses and deductive thematic analyses were performed.

**Results:**

A total of 11 participants responded to the questionnaire and 18 participants to the interviews. Key barriers and facilitators could be mapped onto 6 dimensions, which incorporated aspects of digitization: system use (human), user satisfaction (human), environment (organization), structure (organization), information and service quality (technology), and system quality (technology).

**Conclusions:**

The recommendations proposed can enhance the potential for future remote sensing solutions to be more successfully integrated in health care practice, resulting in more successful use of “virtual wards.”

**Trial Registration:**

ClinicalTrials.gov NCT05321004; https://www.clinicaltrials.gov/study/NCT05321004

## Introduction

Since the onset of the COVID-19 pandemic, adoption and implementation of novel health care pathways have accelerated globally. A key change has been transitioning beyond the traditional face-to-face model of health care delivery with the incorporation of novel remote monitoring solutions [1,2]. They offer a significant advantage in moderating viral exposure risk to health care staff, reducing community spread, and delivering quality health care remotely for exposed or infected individuals [3,4].

The integration of telemedicine and remote monitoring into medical practice is expected to expand by appropriately permitting selected individuals to continue living at home rather than admitting them into secondary care; this very premise is the foundation of “virtual wards” [5]. With the recent improvements made to wearable technology, they can support health provider assessment and clinical decision-making through collected biometric data both in secondary care and in the community [6-10].

However, successful implementation of digital technologies across complex hospital systems is seldom a smooth process [11-13]. The absence of standardized procedures for implementation and evaluation alongside the deficiency of published implementation strategies adds to these difficulties. One study in the National Health Service (NHS) that implemented wearable sensors and alerting systems in secondary care reported no improvements in clinical outcomes among patients [14,15]. The aim was to use wearable sensors to provide continuous remote monitoring to patients admitted to acute (nonintensive) wards and alert health care staff upon recognition of deterioration. Interestingly, although the digital solution was able to pick up clinical deterioration in vital signs and alert health care staff, responding to the alert was met with significant delay. This was in spite of health care staff in the NHS reporting favorable perceptions of digital solutions with potential improvements to patient safety and reduced staff burden [16]. Therefore, there is a need to further explore implementation issues.

Patients have reported high levels of acceptance, comfort, and safety and deemed such digital tools favorable [17-19]. The main concerns, from a patient perspective, surround potential overreliance on numbers with diminishing contact from clinical staff [17,20,21]. Health care staff perceptions, however, have been more mixed, with concerns regarding changing and increasing workloads, uncertainty surrounding the clinical meaningfulness of captured data, and alert fatigue [19-21]. Although mixed methods exploration of these 2 key stakeholder groups has been well documented, understanding how to integrate remote monitoring digital tools in the NHS requires further examination of cultural and management issues in the health care organization, an area where evidence is missing.

In the United Kingdom, large health informatics programs and widespread digital transformations are delivered by NHS Digital, a nondepartmental public body [22,23]. To support digitization, NHS England has formed a framework consisting of 3 ambitions: digital readiness, maturity, and data-enabled services [24]. In line with this, NHS England has supported the development and use of virtual wards, further indicating the “digital push” [5]. For policy makers, understanding the barriers and facilitators as perceived by key organizational members is crucial for the effective provision and smooth deployment of digitally enabled care. A proposed framework evaluates these aspects, incorporating the concept of fit among humans, organizations, and technology (HOT-fit) [25]. This framework offers a structured basis to examine factors that focus on alignment and compatibility across these 3 domains, thereby enhancing the effectiveness of digital health care initiatives.

Therefore, the aim of this study was to evaluate key stakeholder perspectives on an organizational level of implementing remote monitoring solutions in the NHS, identifying factors that could affect successful execution and adoption using the HOT-fit framework. In doing so, we propose a road map for implementing wearable solutions in secondary care.

## Methods

### Study Design

A mixed methods approach was implemented that consisted of semistructured interviews and questionnaires [26]. This was developed in accordance with recommendations from the Standards for Reporting Qualitative Research (SRQR) guidelines where appropriate [27]. The semistructured interviews were conducted with high-level stakeholders from industry and academia, as well as with health care providers who played an instrumental role in and had prior experience of implementing digital solutions. Additionally, a validated questionnaire was used to ascertain the perceived technological acceptance of new remote monitoring systems.

To ensure appropriate recruitment among all key stakeholder groups, a key informant strategy was followed for purposive recruitment [28,29]. Individuals were identified through their notable work with implementation of remote monitoring solutions in health care, including authors of impactful research in the literature, major digital technology companies, technicians involved with digital tool infrastructure development, and experts recommended by peers. This represented a variety of groups, including academics, clinicians, allied health care professionals, and employees of Google Health, who had experience with implementing digital solutions with the NHS.

### Ethical Considerations

All recruited participants provided written informed consent. Ethical approval for this study was obtained by Imperial College London’s Science Engineering Technology Research Ethics Committee (20IC6331), and it was conducted in accordance with the Good Clinical Practice guidelines and the Declaration of Helsinki. Storage and handling of personal data complied with the General Data Protection Regulation. Interviews were recorded, anonymized, and transcribed.

### Questionnaires

**Figure 1:**
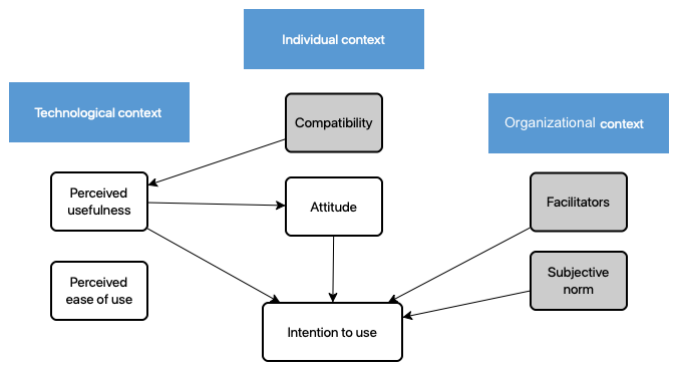
Theoretical framework for the modified Technology Acceptance Model questionnaire [30].

An adapted version of the Technology Acceptance Model (TAM) questionnaire was used; this validated questionnaire has shown acceptably high Cronbach α values [30]. This ensures the reliability of our findings, contributing to the robustness of the study’s methodology and its implications in understanding technology acceptance dynamics. The proposed theoretical framework (information technology acceptance) is shown in Figure 1. It has been adapted from Chau and Hu [31], comprising individual context, technological context, and organizational context. Further adaptations from Gagnon et al [30], with the inclusion of theories of interpersonal behavior and reasoned action building on the TAM, proposed by Davis [32], have been included [30-34]. As such, individual context consists of compatibility (factors that affect acceptance of a new technology) and attitude (perception of the individual to adopting a technology); technological context consists of perceived usefulness and perceived ease of use of technologies. Lastly, organizational context consists of facilitators and subjective norms; the latter can be described as social (an individual’s perception of a behavior) or descriptive (behavior of others).

### Semistructured Interviews

All participants were invited to take part in semistructured interviews conducted by the lead researchers. A structured topic guide was created following a literature review that drew heavily from a model proposed by Simblett et al [35] and by the HOT-fit framework [25].

Data collection was an iterative process; emerging recurring concepts were incorporated into the interview guide for further exploration with remaining participants. Interviews were recorded, anonymized, and transcribed verbatim before being entered into NVivo (version 12; QSR International) for analysis.

### HOT-Fit Framework

**Figure 2:**
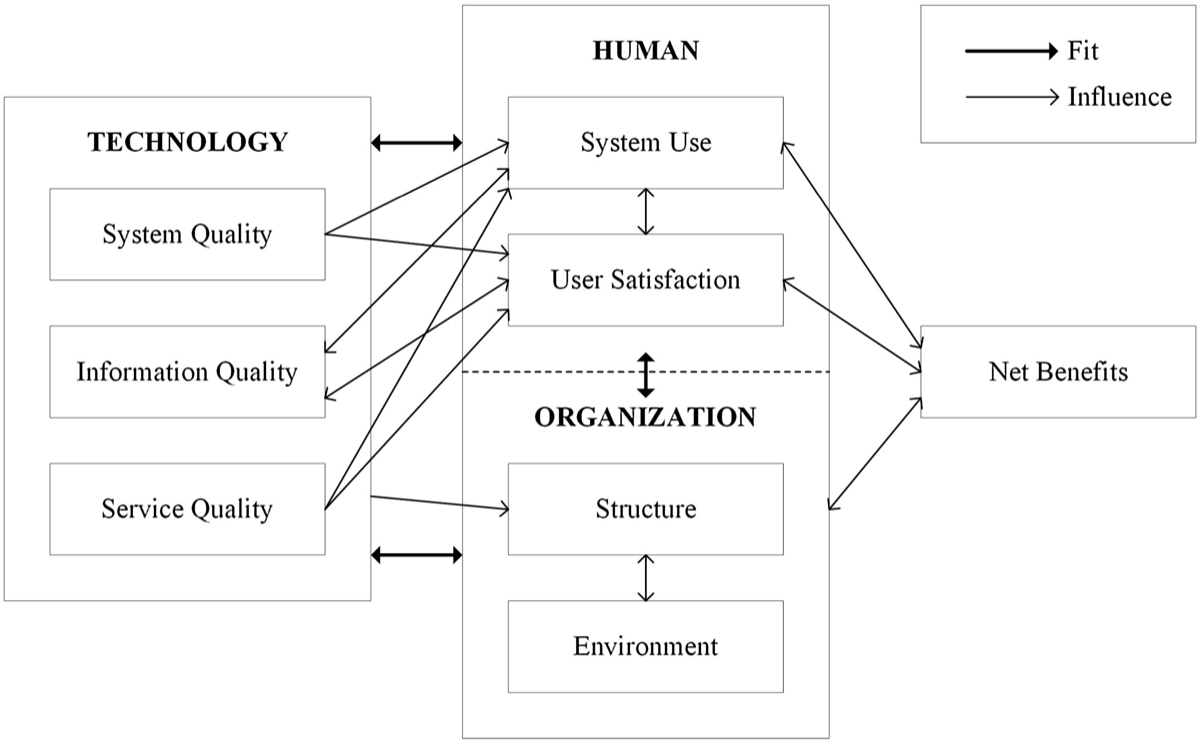
The fit among humans, organizations, and technology (HOT-fit) framework, adapted from Yusof et al [25].

This validated framework identifies dimensions that can be mapped onto and used as reference models for evaluating the performance, effectiveness, and impact of health systems [25,36]. A fit between human, organizational, and technological factors is required to ensure successful implementation and has been highlighted in Figure 2.

### Data Analysis

Frequency distributions were generated for the 7-point Likert scale responses to the modified TAM questionnaire using R studio (R Foundation for Statistical Computing) with the *Likert* package (Bryer and Speerschneider).

Transcribed interviews were analyzed using a broadly deductive approach [37], with the topic guide adapted as previously described [35]. This formed the basis for the initial predefined coding framework and was undertaken by 2 independent researchers to determine barriers and facilitators [37]. An iterative process of coding and data indexing occurred, ensuring key aspects were not missed from the predefined coding framework. Subsequent emerging themes were summarized and mapped to the evaluation measures corresponding to each dimension of the HOT-fit framework [25]. The results were discussed until consensus was reached.

## Results

Overviews of the included participants and the reported evaluation measures are shown in Tables 1 and 2, respectively.

**Table 1:** Demographics of included participants.

Group	Role 1	Role 2	Role 3	Role 4	Role 5	Role 6	Role 7
Health care trusts	Director of strategy, research and innovation	Chief clinical information officer and Caldicott Guardian	Digital quality improvement lead	Project manager	Chief information officer	Systems, integration interoperability architect	Lead nurse for remote monitoring
Academics	Clinical lecturer	Clinical lecturer	Chief scientific advisor	—^a^	—	—	—
Google Health	Clinical lead	Clinical specialist	Product manager	Implementation specialist	Implementation manager	Program manager	—
Other	Programme director: innovation of health	Managing director: digital health	—	—	—	—	—

^a^—Not applicable.

**Table 2:** Overview of reported evaluation measures.

Dimension and evaluation measures			Factors
**System use**			
	Expectation and beliefs	Improved efficiency (facilitator) Appropriate selection of end users suitable for digital tool (facilitator)	
	Training, knowledge, and expertise	Lack of troubleshooting support (barrier) Engagement with new starters (facilitator)	
	Motivation	Large data burden (barrier) Post–COVID-19 fatigue of staff (barrier) Finding local champions (facilitator)	
User satisfaction (no evaluation measures)			Developing relationships for feedback (facilitator) Previous negative experiences with no feedback on benefit (barrier)
Environment (no evaluation measures)			Overburdened National Health Service system (barrier)
Structure (clinical process)			Clear strategic framework and partnership (facilitator)
Information and service quality (no evaluation measures)			Poor interoperability (barrier) Poor user interface and user engagement (barrier)
System quality			Failure to provide added value (barrier)

### TAM Questionnaire

**Figure 3:**
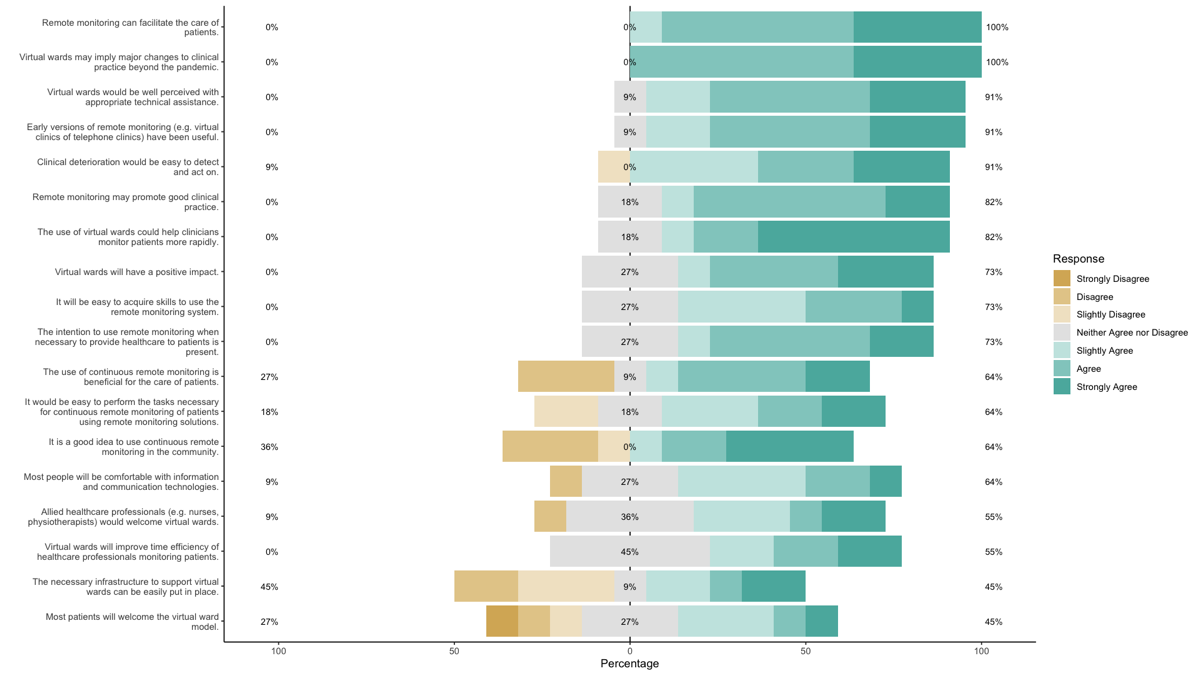
Likert plot displaying responses to the modified Technology Acceptance Model questionnaire. The percentages on the left and right sides of the plot represent the totals for negative and positive responses, while the percentages in the center represent neutral responses.

A total of 11 participants (response rate 11/22, 50%) responded to the questionnaire; the responses are represented as a Likert plot (Figure 3). Overall, the technology surrounding remote monitoring and virtual wards was perceived well by the questioned stakeholders, who considered that it facilitated the care of patients and that these pathways, initially introduced during the pandemic, were likely to change long-term provision of health care. However, some concerns were noted regarding whether the existing infrastructure could support the technology’s use and whether it would improve efficiency. Of note, there was uncertainty regarding whether most patients would welcome virtual wards or remote monitoring.

### Semistructured Interviews

A total of 22 participants were approached, of whom 18 (response rate: 82%) participated in the semistructured interviews (Table 1). An overview of the factors, by dimension, that respondents felt were responsible for contributing to implementation is summarized in Table 2.

#### System Use

##### Expectations and Beliefs

The prospect of introducing novel remote monitoring technologies was felt to facilitate implementation through improved efficiency, particularly since the implementation of electronic health records has improved data availability and clarity:

...with the implementation [and] introduction of electronic health records where the data that’s available is so granular. And in addition to new technologies that are coming. I think that you can do a lot more, remotely or virtually, and it does make things a lot more efficient...

Participant 15

Moreover, respondents also commented that for successful implementation, a selective process should be in place for patients who would benefit the most from novel technologies, rather than using the technology in cases that would not be meaningful:

From a patient perspective, we don’t want to one size fits all approach. We need to be clear about how we personalize this and how it’s relevant and meaningful.

Participant 17

##### Training, Knowledge, and Expertise

Problems with troubleshooting and available training were reported to reduce successful implementation due to a lack of support:

We’ve had problems when trying to use the remote monitoring, it came up with an error and then I have to try and sort that out, you know? It’s just things like that that make extra work.

Participant 16

I know that the nurses have struggled a huge amount with remote monitoring, and I expected that...because there’s a lot of upskilling.

Participant 18

However, engaging early with health care workers and obtaining their involvement was shown to improve implementation of remote monitoring solutions:

[We received] better engagement by tying the implementation with the new starters in the role and the changeover of junior doctors, because it was a new product to offer to new junior doctors.

Participant 3

##### Motivation

It was felt that motivation to engage with technologies would be impacted through the excessive availability of data acting as a deterrent:

We need to be mindful about the data burdens, not just for patients but for staff because this kind of remote technology follows you around. You basically could work 24/7 365 of the year.

Participant 17

In addition, following the pandemic, many health care workers were fatigued and unmotivated to engage in change, acting as a barrier to successful remote technology implementation:

Post-COVID the workforce has been decimated, been exhausted and is fatigued. It’s not the only problem though, because you know as well as I do that the NHS has run this model of where it’s good will. We’ve never had infrastructure that we needed to do stuff and we still get a huge amount done. So it’s not the only driver at the moment. It’s more noticeable because of where people’s heads are at and obviously where their physical levels and mental levels of exhaustion are...

Participant 17

However, respondents also noted that finding a few motivated individuals to champion change at a local level can help implementation:

I asked them to self-nominate three of them who were interested in helping [implement]. So they led and supported the [technology]...

Participant 18

#### User Satisfaction

Respondents reported that previous experience with digital tools tied into user satisfaction. Feedback to end users demonstrating meaningful impact was deemed important for engagement and successful implementation:

Where staff or patients, for example, have been involved in projects before that they haven’t had any feedback from, haven’t seen any meaningful outcome from...they’re like, well, why would I want to get engaged with this? That’s a lot of energy and effort from me and I won’t see any benefit.

Participant 17

...develop relationships, so between, if you like, supplier and developer and clinical staff so you’ve got these rapid cycles of feedback and learning.

Participant 1

#### Environment

Respondents reported that previous hindrance of effective implementation was because of an overburdened system unable to give the appropriate attention to integrating a digital solution in the NHS:

NHS is overburdened and so that level of diligence...wasn’t there until it had to be, until things became mission critical...that comes down to a bandwidth problem...

Participant 10

Similarly, underresourcing was noted to be a barrier, particularly during the early stages, where issues would arise:

More resource[s] to get [things] kick started [are usually needed]...because we had to go through all the teething problems ourselves which created extra work for us.

Participant 16

We’ve got very limited resources, that they’re very thinly spread across all of the IT projects that require integration and interoperability...just the sheer volume of work that the Trust has heaped on us over the last three or four years is the bigger constraining factor.

Participant 4

Lastly, organizational culture supporting digitization was a commonly reported theme, with some institutions more readily accepting of innovation than others:

Organisational culture can be both the barrier and facilitator. We know that there are some organisations that are much more ready and able to adopt innovation. I think from an organisational perspective, competing priorities are a huge issue...If your IT is majorly engaged in doing something else, for example an EHR implementation, its ability to support remote monitoring and other technologies is really poor.

Participant 17

#### Structure

Respondents also commented on the need for a clear process and said that developing a strategic partnership and framework would facilitate implementation and should be planned before rollout:

Strategic framework is crucial on things.... What does a strategic partnership look like? What is the direction that we want to jointly head in? What do we want to achieve together, and what are the different components to get there...

Participant 1

Making [the product vision and roadmap] clear as early on and getting that input right at the beginning of any kind of feature development. So that there is expectation alignment on what is being developed whether the minimal viable product meets the use cases that it needs to, and that there’s a partnership in prioritizing these features and when they’re delivered. As opposed to just showing a feature set a few weeks before it gets deployed.... I think that initial understanding of the vision...and getting that clinical engagement as early on helps to set the path going forward.

Participant 12

#### Information and Service Quality

Respondents noted the need for digital tools to be interoperable and usable, as poorly designed digital tools would be a barrier, hindering an overly strained NHS system:

The challenges are IT and interoperability…you don’t want 20 bits of data…from 20 apps that don’t work, so that’s the usability and the accessibility and the staffing of these models because traditionally they basically get added onto someone’s day job. But that day person’s already overwhelmed.

Participant 17

#### System Quality

Respondents highlighted that for a digital tool to be successfully implemented, it needed to provide added value, with perceived usefulness and ease of use being crucial.

[What] was the added value in [this digital app]? All it did was render some of the information that we already had in a limited manner, back in the mobile device.

Participant 8

Usability, the accessibility, and the staffing of these models [are really poor] because traditionally they basically get added onto someone’s day job.... The data element [is also] really poor, so you get a lot of enthusiasts doing a lot of projects. But if you then say where’s your evidence that makes any difference to anything meaningful that matters to patients and staff, they can’t produce that. I think the digital health tech industry has been really slow at that.

Participant 17

Furthermore, it was believed that the best way to implement a digital tool (eg, remote monitoring solutions) was through rapid quality improvement cycles following the plan-do-study-act (PDSA) technique, focusing on targeting user experience issues:

...believe the technology suffered from very poor clinical and user engagement. So I know [technological companies] will tell us they’ve had loads of user engagement, but actually most patients wouldn’t say that, they’d say well, why is it like this? No, why is nobody been engaged in the design for this?

Participant 17

...trying to give clinical input into feasibility, usability, implementation in terms of the design of how we were going to implement stuff, so...[a] genuine PDSA type approach to implementation, and I was quite involved in some of the thinking about spread and how do you get this utilized across different parts of the Trust...

Participant 1

## Discussion

### Principal Findings

This study explored barriers and facilitators for implementing digital tools, in particular remote monitoring solutions, in the NHS, alongside the acceptance of such technology using the modified TAM questionnaire. Using the HOT-fit framework, human, organization, and technological factors were categorized, allowing for a multiple-angled approach to a multifaceted problem. Therefore, key barriers and facilitators could be mapped onto 6 dimensions, which incorporated aspects of digitization: system use (human), user satisfaction (human), environment (organization), structure (organization), information and service quality (technology), and system quality (technology).

With regards to system use, the importance of improving workflow efficiency, having appropriate troubleshooting support available for staff, finding local champions to help integration within the clinical workforce, and positively engaging with health care staff were highlighted as facilitators. To support this, young staff have been deemed the most likely to engage with and benefit from a new workflow [36,38-40]. This, in part, may be explained by more adept digital literacy skills and technical proficiencies associated with junior members [41]. In the literature, concise and tailored education surrounding implementation has been promoted as an important facilitator [42].

Key barriers relating to system use and environment included poor training and the burden of data, particularly with continuous remote monitoring of vital signs. These data may not always be clinically meaningful or because of poor resourcing may not be acknowledged appropriately, generating additional work for existing staff, who are already overburdened [14,43]. Previously, this unincentivized workflow change led to poor response times to alerts generated through alerting systems in an acute surgical ward [14]. In this study, 36% (4/11) of respondents to the modified TAM questionnaire were unsure whether allied health care professionals would welcome virtual wards (Figure 3). One study highlighted that these workers, in particular nurses and clinicians, were the most important gatekeepers for remote monitoring solutions [44]. Therefore, engaging these groups, fostering positive relationships, and delivering regular feedback would enhance user satisfaction, allow user interface and engagement issues to be proactively tackled, and subsequently enable successful implementation.

Concerning system quality, perceived usefulness and ease of use were deemed as important facilitators for successful implementation. In the literature, intuitive and user-friendly systems have been confirmed to have easier acceptance [36,39]. The modified TAM questionnaire similarly confirmed this in our cohort, particularly through questions concerning acquiring new skills and impact, emphasizing that remote monitoring technology could be readily accepted.

### Limitations

This study included key stakeholders belonging to a broad selection of groups (academics, industry, and health care) in order to create a broad understanding of factors that influence implementation of remote monitoring solutions in the NHS. Given that previous studies have focused on end user testing, this study sought to provide a top-down view to give a better understanding of considerations that could influence widespread implementation [16,18]. However, in doing so, our interpretations have some limitations. First, the broad, heterogeneous sample of key stakeholders included may identify issues that are generalizable, but the nonprobabilistic sampling may have resulted in a selection bias. Moreover, the included sample size was limited. Despite this, the use of semistructured interviews yielded pertinent considerations for pragmatic implementation in hospital settings. In addition, differences between various hospitals and departments, which may have different attitudes toward digital technologies, were not explored in this study. A final limitation relates to the HOT-fit framework; although it is considered useful, the mapping of factors is a subjective undertaking and mapping to one specific measure was, at times, difficult.

### Further Research and Recommendations

Although our cohort showed that there was overall acceptance of remote monitoring technology (Figure 3), there remains a deficiency with respect to successful implementation. This was noted most recently in one study where the median time to acknowledge an alert from health care staff was 111 (range 1-2146) minutes, despite early recognition of deterioration from remote sensing [14]. Therefore, further research should incorporate human factors and behavior evaluation when implementing remote monitoring solutions with the NHS; moreover, implementation frameworks such as HOT-fit should be used to ensure multiple angles have been carefully considered.

To facilitate the effective integration of remote monitoring solutions in clinical workflows, a comprehensive strategic framework is paramount. This framework should prioritize the early involvement of end users, fostering relationships that enable rapid feedback on implementation strategies, user interfaces, and user experience issues. Such engagement allows for iterative enhancements through PDSA cycles, promoting continuous improvement [45].

Industries aiming to develop remote monitoring technologies must collaborate closely with key stakeholders, ensuring the creation of products that provide significant value and feature user-friendly interfaces. This approach emphasizes the importance of a bottom-up strategy in technology implementation, valuing the autonomy and insights of end users, who play a crucial role in the successful adoption of these solutions. Crucial to this process is the establishment of robust infrastructural support prior to the deployment of remote monitoring systems. Adequate resourcing and the involvement of technical support staff are essential to facilitate seamless integration with existing information technology frameworks, thereby enhancing the prioritization and effectiveness of digital health initiatives. By adhering to these guidelines, health care organizations can enhance the integration of remote monitoring into clinical practice, leading to improved operational efficiency, patient care, and overall health care service delivery.

### Conclusion

Implementation of remote monitoring solutions in the NHS remains a complex challenge. The results of this study have highlighted key stakeholder perceptions that could influence successful integration. Through the proposed recommendations, there is potential for future remote sensing solutions to be more successfully integrated into our health care practices, resulting in novel pathways expanding beyond virtual wards.

## Abbreviations

HOT-fit: fit among humans, organizations, and technology
NHS: National Health Service
PDSA: plan-do-study-act
SRQR: Standards for Reporting Qualitative Research
TAM: Technology Acceptance Model

## Data Availability

The data sets used and/or analyzed during the study are available from the corresponding author on reasonable request.
